# Calibration of Low-Cost NO_2_ Sensors through Environmental Factor Correction

**DOI:** 10.3390/toxics9110281

**Published:** 2021-10-28

**Authors:** Jason A. Miech, Levi Stanton, Meiling Gao, Paolo Micalizzi, Joshua Uebelherr, Pierre Herckes, Matthew P. Fraser

**Affiliations:** 1 School of Molecular Sciences, Arizona State University, Tempe, AZ 85287, USA; jmiech@asu.edu (J.A.M.); Pierre.Herckes@asu.edu (P.H.); 2 Clarity Movement Co., Berkeley, CA 94710, USA; levi@clarity.io (L.S.); meiling@clarity.io (M.G.); paolo@clarity.io (P.M.); 3 Maricopa County Air Quality Department, Phoenix, AZ 85012, USA; Joshua.Uebelherr@maricopa.gov; 4 School of Sustainable Engineering and the Built Environment, Arizona State University, Tempe, AZ 85287, USA

**Keywords:** nitrogen dioxide, low-cost sensors, ozone, air quality

## Abstract

Low-cost air quality sensors (LCSs) have become more widespread due to their low cost and increased capabilities; however, to supplement more traditional air quality networks, the performance of these LCSs needs to be validated. This study focused on NO_2_ measurements from eight Clarity Node-S sensors and used various environmental factors to calibrate the LCSs. To validate the calibration performance, we calculated the root-mean-square error (RMSE), mean absolute error (MAE), R^2^, and slope compared to reference measurements. Raw results from six of these sensors were comparable to those reported for other NO_2_ LCSs; however, two of the evaluated LCSs had RMSE values ~20 ppb higher than the other six LCSs. By applying a sensor-specific calibration that corrects for relative humidity, temperature, and ozone, this discrepancy was mitigated. In addition, this calibration improved the RMSE, MAE, R^2^, and slope of all eight LCS compared to the raw data. It should be noted that relatively stable environmental conditions over the course of the LCS deployment period benefited calibration performance over time. These results demonstrate the importance of developing LCS calibration models for individual sensors that consider pertinent environmental factors.

## 1. Introduction

Although annual ambient NO_2_ concentrations have been decreasing in many locations in North America, many East Asian countries are seeing increases in the annual mean NO_2_ concentration [1]. Studies have shown that exposure to NO_2_ can lead to increased preterm birth and infant mortality, increased asthma symptoms, allergic rhinitis, and chronic obstructive pulmonary diseases [2,3]. The U.S Environmental Protection Agency’s (EPA) current National Ambient Air Quality Standard (NAAQS) for 1-h NO_2_ is 100 ppbV, while the World Health Organization’s 2005 1-h NO_2_ guideline value is 200 μg/m^3^ [4,5]. Even in areas that meet health-based standards to protect against exposure to NO_2_, there is still the need to measure ambient NO_2_ levels, as photochemical reactions of NO_2_ can result in ground-level ozone and fine particle formation [4]. Anthropogenic NO_2_ is mainly formed through high-temperature combustion processes from both mobile and stationary sources; the EPA’s 2017 National Emission Inventory reports 52% of NO_2_ emissions from mobile sources such as cars, trucks, and planes, and 32% from stationary sources such as power plants and cement kilns [6,7].

The main purpose of air monitoring networks is to observe the community’s exposure to air pollutants. In Maricopa County, Arizona, regulatory monitoring includes quantification of ambient levels of NO_2_, CO, PM_10_ (particulate matter with an aerodynamic diameter ≤ 10 µm), PM_2.5_ (particulate matter with an aerodynamic diameter ≤ 2.5 μm), SO_2_, and O_3_. This is accomplished by using 29 air monitoring stations spread out across the county that utilize EPA-approved federal reference methods (FRMs) and federal equivalent methods (FEMs), are regularly calibrated, and produce high-quality data. However, not all of these stations measure all regulated pollutants, and the FRM instruments require expensive sensing equipment and intensive calibration and verification. As a result of the comparably high FRM cost of installation and operation, the limited number of stations measuring each pollutant provides a relatively coarse spatial resolution and community coverage, as compared to finer-scale low-cost air quality sensors (LCSs) deployed in a county as large as Maricopa County. For example, the NO_2_ FRM at West Phoenix collects data, defined by the monitor’s surrounding Thiessen polygon, that is expected to represent the exposure to that pollutant over an area of 1278 km^2^, which is home to over 1 million people [8]. Zhu et al. demonstrated the importance of increasing spatial coverage of NO_2_ monitoring, as often these government monitoring stations do not properly cover pollution hot spots [9].

An alternative approach to air monitoring instrumentation are LCSs, which have recently become more popular as their capabilities increase and cost decreases. Often, these sensors can measure several pollutants simultaneously, require minimal setup, can be easily moved, and can cost less than two thousand dollars. These sensors can be used to study small-scale processes or supplement larger air monitoring networks by increasing their spatial resolution [10,11,12]. However, these sensors have been shown to be less accurate and precise compared to FRMs, and often require collocation with a FRM to develop a calibration model [13,14]. In a 2020 report, the U.S. Government Accountability Office highlighted challenges that the nation’s air quality monitoring system is facing, including aging infrastructure and decreasing funding, and recommended a modernization plan that includes further study on the performance and calibration of LCSs [15].

Although the EPA has published Performance Targets and Testing Protocols of LCSs for PM_2.5_ and O_3_, it has yet to publish guidance on the appropriate approach to evaluate NO_2_ LCSs [16,17]. However, the South Coast Air Quality Management District (SCAQMD) has tested a variety of NO_2_ LCSs and publishes its results online [18]. Table 1 summarizes the testing results of NO_2_ measured by LCSs that use electrochemical sensors.

While SCAQMD only tests LCSs as-is, there are a variety of published studies that have tested various calibration techniques on similar sensors. Han et al. [20] conducted a 12-month field evaluation of four Alphasense NO2-B43F sensors and tested several linear regression and neural network calibration methods that accounted for temperature and relative humidity. They concluded that the neural network significantly improved the NO_2_ data compared to the other methods. However, one potential problem in this study is that they used one set of sensors to train the calibration models and the other set to test sensor performance, resulting in questions about the performance between individual LCSs deploying the same sensing technology. Suriano et al. [21] designed and tested a field evaluation system for LCSs, including two Alphasense NO2-B43F sensors, and used linear regression and multivariate linear regression models to calibrate these sensors, considering the interfering effects of relative humidity, temperature, and ozone. Sahu et al. [22] also evaluated Alphasense NO2-B43F sensors and recommended incorporating temperature and relative humidity into calibration models and maximizing the diversity of data used in training; they also concluded that using a local non-parametric calibration method with a learned metric maximized sensor performance. Another study, Masey et al. [23], tested Aeroqual S5000 NO_2_ sensors and used a linear regression calibration model that included ozone but not temperature and relative humidity. This study analyzed the impact of varying training datasets on calibration performance and concluded that the best performance came from combining data from intermittent periods during the deployment into one training dataset. Lin et al. [24] further studied the impact of ozone on Aeroqual S5000 NO_2_ sensors and found that the sensor bias was significantly correlated to nearby ozone measurements and saw improved sensor performance after correcting for ozone.

Despite these prior studies, questions remain about the intercomparability of multiple LCSs measuring NO_2_ using the same sensing approach. In this work, we evaluated the performance of eight NO_2_ LCSs of the same make and model compared to a FRM instrument over a 4-month period. The number of LCSs used allowed us to better characterize their performance as a cohesive network and identify any outliers. We examined the impact of environmental factors such as temperature, relative humidity, and ozone on the LCSs and developed calibration methods to correct for these potential influences. Additionally, we studied the impact of training data volume on the performance of the calibration, in terms of accuracy and precision among the LCSs.

## 2. Materials and Methods

### 2.1. Low-Cost Sensors and Reference Monitoring

The collocation study was conducted in Maricopa County, Arizona, USA for 4 months, from October 2020 to February 2021. One of the largest urban areas in the Southwestern United States, Maricopa County has a growing population of 4.4 million residents with elevated levels of air pollution, exceeding the EPA’s 8-h O_3_ standard of 70 ppb [25]. Over the study period, the meteorological conditions varied widely, with a temperature range of 2–34 °C and a relative humidity range of 6–97%. Eight LCSs, Clarity Node-S models (Clarity Movement Co., Berkeley, CA, USA), were used in this study. These devices are capable of measuring PM_2.5_ and NO_2_ while also containing sensors to track the temperature and relative humidity inside the device. The NO_2_ measurement is made with an Alphasense NO_2_-A43F electrochemical cell (Alphasense Ltd., Great Notely, UK) that has an ozone filter at the front end to limit interfering species, as the electrochemical cell has a 100% cross-sensitivity with ozone [26]. These LCSs are solar powered and take and report measurements every 15 min, uploading these data to the Clarity cloud using a cellular connection, where the data are then averaged into hourly measurements.

**Figure 1:**
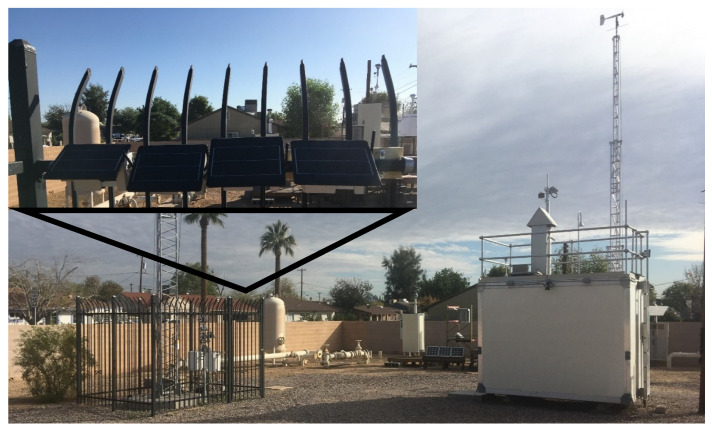
West Phoenix Air Monitoring Site with the Clarity Node-S LCS.

These LCSs were collocated with a NO_2_ FRM instrument at Maricopa County Air Quality Department’s West Phoenix monitoring site (AQS ID: 04-013-0019). This air monitoring site operates at a neighborhood scale (radius of 0.5–4 km) while covering a population of over one million individuals [8]. The LCSs were mounted on a fence 2 m above the ground and 10 m away from the FRM inlet as shown in Figure 1. The NO_2_ FRM instrument at this site is a Thermo Scientific 42iQ NO-NO_2_-NO_x_ Analyzer (ThermoFisher Scientific, Franklin, MA, USA), while the O_3_ FEM instrument is a Teledyne-API T400 (Teledyne API, San Diego, CA, USA). Data from these instruments were obtained from MCAQD at a 1-h resolution.

### 2.2. Calibration Models

Three data series are compared in this study; the raw performance of the sensors based solely on NO_2_ sensor response; a calibration model applied by the manufacturer to correct bias and account for sensor response to changing temperature and relative humidity; and a calibration model that starts with the manufacturer calibration that further accounts for sensor response to changing ozone concentrations. The training and testing time periods of these calibration models were varied in an attempt to optimize their performance.

#### 2.2.1. Raw Calibration

Raw NO_2_ concentrations are calculated by Clarity using the potentiostat voltages proportional to the working and auxiliary electrodes (vGas and vAux, respectively) measured from an Alphasense NO_2_-A43F sensor using Equation (1), where asSensitivity is a sensor-specific value provided by the manufacturer that allows for the conversion from nA to ppb. The 10^6^/499 quantity is specific to the implementation of the potentiostatic circuit and is calculated by solving the circuit (in particular, the current measuring circuit).

(1) $${\mathrm{NO}}_{2}{}_{\mathrm{Raw}}=\frac{\left(\mathrm{vGas}-\mathrm{vAux}\right)*\left(\frac{10^{6}}{499}\right)}{\mathrm{as}\;\mathrm{Sensitivity}}$$

#### 2.2.2. Clarity Baseline Calibration Model

The Clarity baseline calibration model uses the electrochemical cell’s working and auxiliary voltage along with the sensor’s internal temperature and relative humidity readings in a multivariate regression model. This model is fit to collocation data for each sensor, which corrects for shifts in the baseline caused by the current temperature and relative humidity changes in the recent past. This calibration model was then compared against the NO_2_ measurement by the FRM sensor.

#### 2.2.3. Ozone Correction Calibration Model

**Figure 2:**
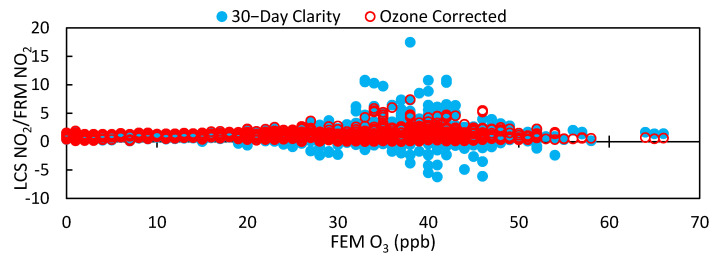
Ratio of LCS NO_2_ values to FRM NO_2_ values vs. FEM O_3_ for the Clarity 30-day calibration and the Ozone corrected 30-day calibration.

Initial review of data showed a high correlation between the LCS NO_2_ measurements and FEM O_3_ measurements as shown in Table S1. To elucidate the impact of ozone on NO_2_ measurements, a second calibration model, Equation (2), was applied on top of the Clarity baseline calibration to also incorporate LCS response to changing ozone concentrations. We proceeded with this approach as O_3_ FEM measurements were available at the West Phoenix collocation site, and in future field deployments, these LCSs will also be collocated with O_3_ FEM instruments. Figure 2 demonstrates that ozone only has a noticeable effect on the LCS NO_2_ measurements above a certain threshold value, *a*, which is reflected in Equation (2). Lab studies performed on the Alphasense NO2-B43F, which has a larger ozone filter than the NO_2_-A43F model, have shown an O_3_ cross-sensitivity of 6.6% and warned that this cross-sensitivity could increase over time [27]. This same study found that the NO2-B43F signal was linearly dependent on relative humidity with hysteresis and that six of the LCSs had a direct relationship between temperature and reported NO_2_ concentrations, but that two sensors showed an inverse relationship between sensor values and temperature. This study was unable to explain this discrepancy and may indicate that LCSs built from the same components may behave differently in field deployments.

The Clarity baseline calibration model used in this study can interpret low NO_2_ concentrations as zero or negative concentrations; however, when viewed by consumers, the negative concentrations are clipped. The ozone correction calibration model applies a second correction to force all data above zero, as detailed in Equations (3)–(5).

(2) $$x=[\left(O_{3}>a)\to ({\mathrm{NO}}_{2_{\mathrm{Clarity}}}-\left(c_{1}*O_{3}\right)\right)\wedge (\neg (O_{3}>a)\to {\mathrm{NO}}_{2_{\mathrm{Clarity}}})]+c_{2}$$

(3) $$y=(x<0)\to (x+(-c_{3}*x))\wedge (\neg (x<0)\to x)$$

(4) $$z=\left((1<y<b)\to \left(y+\frac{c_{4}}{y}\right)\right)\wedge \left(\neg \left(1<y<b\right)\to y\right)$$

(5) $${\mathrm{NO}}_{2_{\mathrm{Ozone}\;\mathrm{Corrected}}}=((0<z<1)\to (z+c_{5}*z))\wedge (\neg (0<z<1)\to z)$$

### 2.3. Calibration Evaluation Methods

From the LCS NO_2_ data, several parameters, including root-mean-square error (RMSE), mean absolute error (MAE), R^2^, standard deviation, and slope were calculated and used to evaluate the performance of each calibration compared to the FRM NO_2_ measurements. RMSE and MAE were used to quantify the accuracy of the LCSs, R^2^ was used to quantify the fit of the data relative to FRM measurements, and standard deviation was used to determine precision among the LCSs. Additionally, slope and the Pearson correlation coefficient were used to quantify the impact of environmental factors such as relative humidity, temperature, and ozone on the LCSs. RMSE was calculated using the procedure documented in the EPA’s Performance Testing Protocols, Metrics, and Target Values for O_3_ Air Sensors; MAE was calculated using Equation (6), where *y_i_* is the LCS measurement and *x_i_* is the FRM measurement; and standard deviation was calculated using Equation (7) [17].

(6) $$\mathrm{MAE}=\frac{\sum_{i=1}^{n}\left|y_{i}-x_{i}\right|}{n}$$

(7) $$\mathrm{SD}=\sqrt{\frac{\sum_{i=1}^{n}{\left(y_{i}-\bar{y}\right)}^{2}}{n-1}}$$

## 3. Results and Discussion

### 3.1. Raw LCS NO_2_ Measurements

**Figure 3:**
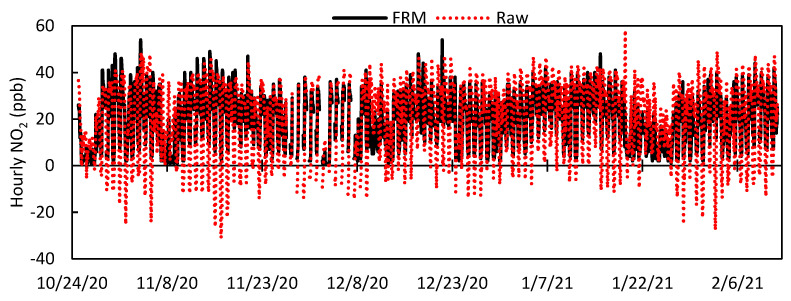
Time series of hourly West Phoenix FRM NO_2_ and LCS #2 Raw NO_2_ values for the entire deployment period.

**Figure 4:**
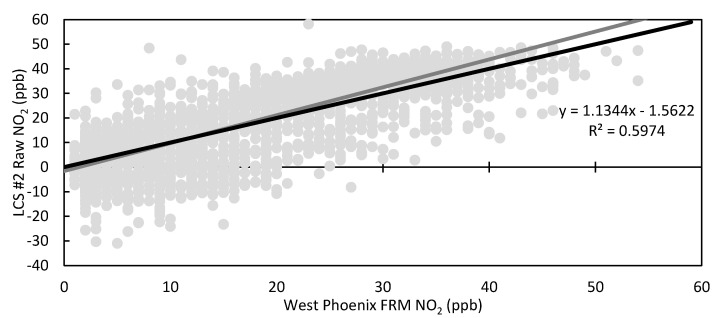
Scatter plot of hourly LCS #2 Raw NO_2_ vs. West Phoenix FRM NO_2_ with trendline equation and R^2^, 1:1 line in black.

Figure 3 shows the times series plots for the West Phoenix NO_2_ FRM instrument and the raw data from LCS #2, while Figure 4 is a scatter plot of LCS #2 raw NO_2_ vs. West Phoenix NO_2_ FRM with linear regression parameters. In Figure 3, it is apparent that the raw LCS data under-measure NO_2_ compared to the FRM and regularly report negative values. Figure 4 indicates that the overall responsiveness of the LCSs reflects changing NO_2_ concentrations as the slope is close to 1; however, variability, as demonstrated by the data scatter, shows LCS shortcomings. Statistics for the colocation period from all eight LCSs are shown in Table 2. By testing eight LCSs, we demonstrated that not all sensors of the same make and model always behave the same, as we saw that LCS #5 and #11 have substantially larger RMSE values and lower R^2^ values than the other LCSs. By including the performance of these two outlier LCSs, the standard deviation between all LCSs for the whole period was 8.8 ppb; however, excluding the two outlier LCSs resulted in the standard deviation dropping to 3.2 ppb. Results from the other six LCSs fell in the range of previously studied LCSs shown in Table 1; for example, Sensor 2 had a MAE of 8.7 ppb and a R^2^ of 0.5974, which were comparable to AQMesh (V5.1) LCS.

**Figure 5:**
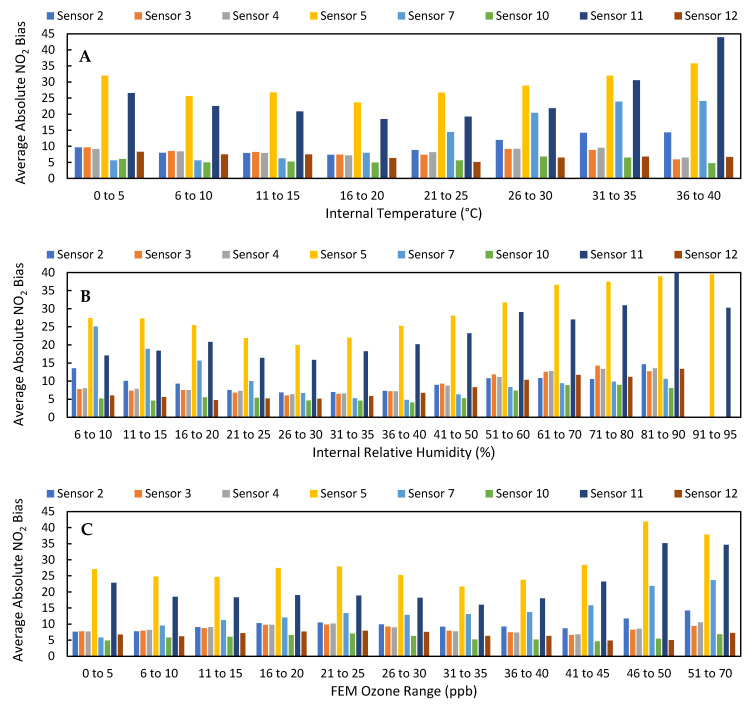
(**A**) The average absolute bias of the raw LCS NO_2_ data binned by internal temperature values. (**B**) The average absolute bias of the raw LCS NO_2_ data binned by internal relative humidity values. (**C**) The average absolute bias of the raw LCS NO_2_ data binned by West Phoenix FEM O_3_ values.

As shown in numerous other studies [20,21,22,23,24], the data from LCSs can be improved through calibrations that account for relative humidity, temperature, and ozone, among other factors. Table S1 provides evidence that the raw output from these sensors is affected by these environmental conditions compared to their effect on the FRM instrument. In Table S1, we see that the raw LCS NO_2_ data was more strongly impacted by environmental parameters such as relative humidity, temperature, and ozone; LCS #2 had a higher Pearson correlation to temperature, relative humidity, and ozone (T: −0.66, RH: 0.52, O_3_: −0.89) than the FRM data (T: −0.32, RH: 0.14, O_3_: −0.81). This effect is demonstrated in Figure 5, as the average absolute biases of the LCSs were higher when the relative humidity was greater than 35% and ozone was greater than 40 ppb. Additionally, Figure 5 and Table S1 further highlight the difference between LCSs #5 and #11 and the rest of the sensors, as they typically had absolute biases 20 ppb higher and slopes double those of the other sensors.

### 3.2. Clarity LCS NO_2_ Calibration

**Figure 6:**
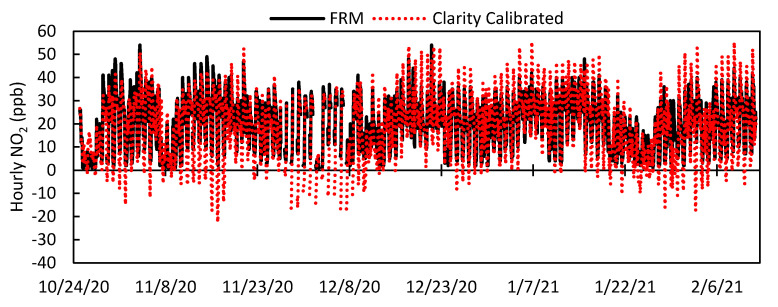
Time series of hourly West Phoenix FRM NO_2_ and LCS #2 Clarity 15−Day calibration NO_2_ values for the entire deployment period.

**Figure 7:**
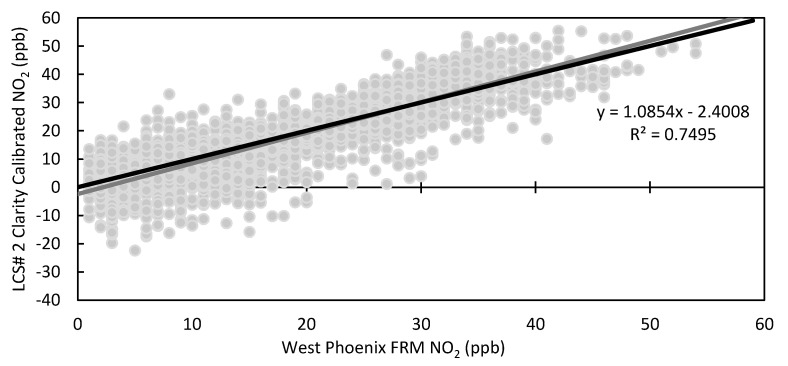
Scatter plot of hourly LCS #2 Clarity 15-day calibration NO_2_ vs. West Phoenix FRM NO_2_ with trendline equation and R^2^, 1:1 line in black.

**Figure 8:**
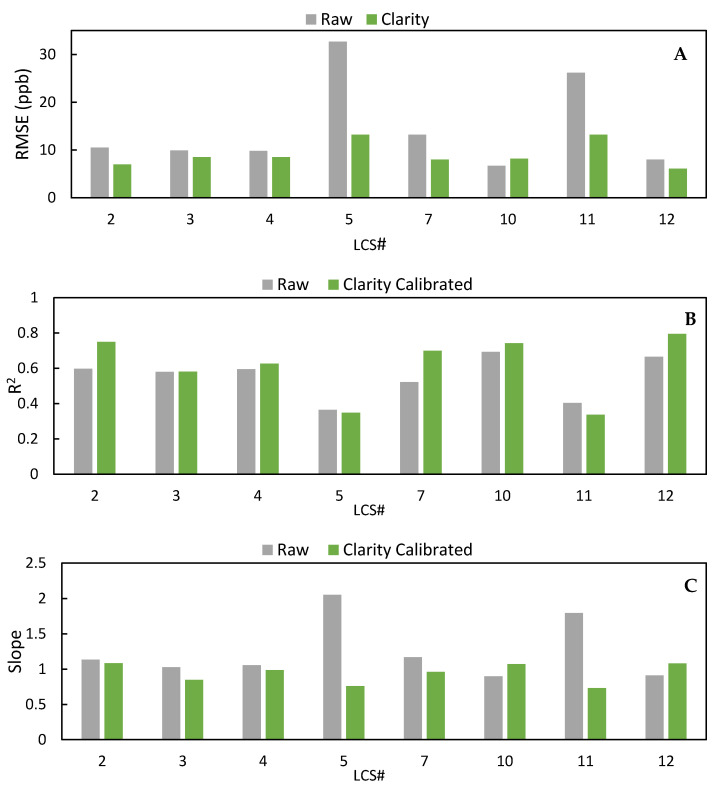
(**A**) A comparison of the RMSE values (ppb) between the raw and Clarity 15-day calibrated data. (**B**) A comparison of the R2 values between the raw and Clarity 15-day calibrated data. (**C**) A comparison of the slopes between the raw and Clarity 15-day calibrated data.

To correct for varying responses to environmental conditions, Clarity applies a calibration to the raw NO_2_ concentration that accounts for the impact of temperature and relative humidity on sensor response. To develop their calibration, Clarity recommends collocating their LCSs with a FRM/FEM instrument for at least 2 weeks. This initial calibration was trained using data from a 15-day period (26 October 2020–9 November 2020 LCS #2 *n* = 361). The time series for the LCS #2 calibrated data is seen in Figure 6 with the scatter plot in Figure 7. After the application of the correction calibration, the LCS data underestimated and overestimated NO_2_ at times with a decrease in the number of negative values (*n* = 226) compared to the raw data (*n* = 366). Summarized statistical results of all sensors for the whole deployment period are in Table 3, while Figure 8 directly compares the raw data to the Clarity calibrated data. Figure 8A clearly shows that the calibration reduced the RMSE from an average of 15 ppb to 9 ppb, and Figure S1 shows a reduction in MAE from 12 ppb to 7 ppb for all LCSs. Additionally, the average standard deviation over the whole deployment period was lowered from 8.8 ppb to 5.5 ppb. However, the scatter in the data was not uniformly improved, as LCS #5 and #11 had lower R^2^ values (0.35, 0.34) with the Clarity calibration compared to the raw data (0.36, 0.40) (Figure 8B). In terms of slope, the Clarity calibration improved LCS #5 (2.1 to 0.76) and #11 (1.8 to 0.73) with minimal improvements seen in the other sensors (Figure 8C). Additionally, as seen in Figure 6 and Figure 7, the calibration did not completely remove negative or zero values of NO_2_, leading to larger errors.

**Figure 9:**
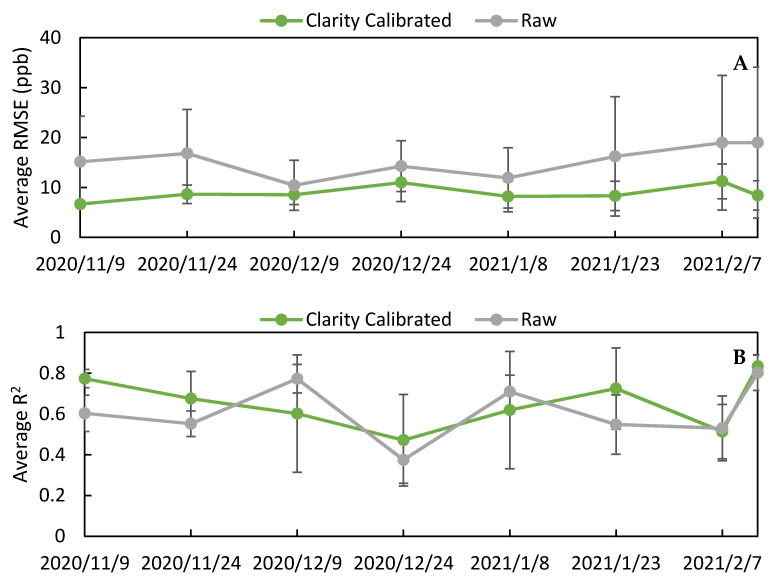
Average RMSE (**A**) and average R^2^ (**B**) between the 8 sensors evaluated biweekly for the whole deployment period for raw NO_2_ and Clarity 15-day calibration.

In addition to comparing raw and Clarity calibrated data across the whole period, we wanted to compare data excluding the calibration training period and evaluate how the calibration performed over time. By excluding data from the calibration training period during the first 15 days of deployment, we were able to demonstrate the predictive ability of the calibration model. When this analysis was performed (Figure S2), we saw the same trends as when the whole period was evaluated: specifically, a uniform decrease in RMSE and MAE and minimal changes in R^2^ and slope, indicating that the calibration did improve the data during the test period. To evaluate the calibration over time, the RMSE and R^2^ values for the LCSs were calculated for 2-week periods, as shown in Figure 9 for both the raw and Clarity calibrated data, to produce a temporal trend. This analysis showed no statistical change in sensor performance over time. This lack of sensor drift can be explained by looking at the environmental parameters experienced by the LCSs over their deployment period. Figure S3 shows that temperature, relative humidity, NO_2_, and O_3_ did not substantially differ between the calibration period and the final 15 days of deployment (29 January 2021–12 February 2021), indicating that the training data spanned the range of environmental conditions experienced during the study.

### 3.3. Impact of Volume of Training Data on Calibration Performance

**Figure 10:**
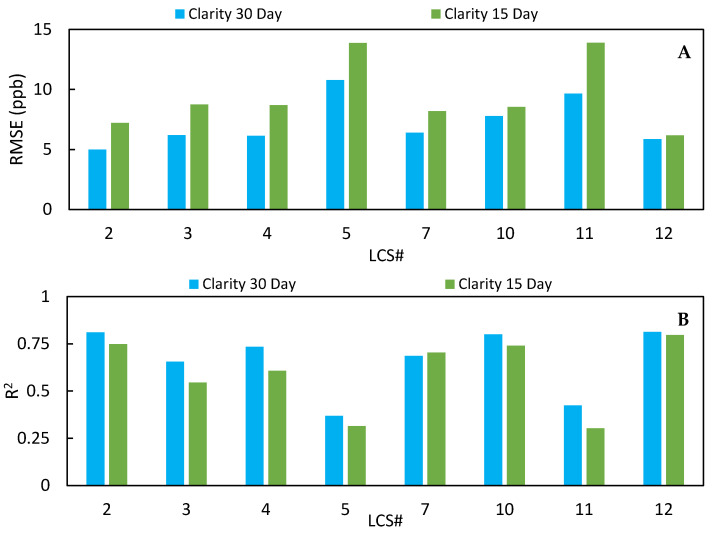
Average RMSE (**A**) and average R^2^ (**B**) of the 8 LCSs for the Clarity 30-day and Clarity 15-day calibration from the test period (24 November 2020–12 February 2021).

To investigate possible approaches to improve the calibration process, we tested whether larger training data volume resulted in an improvement of the LCS measurements. For this, we doubled the volume of data the Clarity model was trained on to include data collected between 26 October 2020 and 23 November 2020 (LCS #2 n=721). Figure 10 shows the RMSE (A) and R^2^ (B) values of the test period for the Clarity calibrated data using 15- and 30-day training periods. By using ~30 days for calibration, the RMSE decreased for all sensors during the test period, there was less scatter in the data except for LCS #7, and the standard deviation decreased to 4.3 ppb. Table 4 and Table S2 summarize the results of the 30-day Clarity calibration for the whole period, including how the data responded to environmental conditions. The 30-day Clarity calibrated data, Table S2, better accounted for environmental biases in the LCSs relative to the NO_2_ FRM measurement compared to the raw data previously shown in Table S1. For example, LCS #2 with the 30-day Clarity calibration had similar correlations to temperature, relative humidity, and ozone (T: −0.30, RH: 0.03, O_3_: −0.73) compared to the FRM data (T: −0.32, RH: 0.14, O_3_: −0.81). The 30-day Clarity calibrated data also had higher correlation coefficients with the FRM data (0.66 ≤ x ≤ 0.89) compared to the raw data (0.60 ≤ x ≤ 0.83). However, the 30-day Clarity calibration only adjusted for temperature and relative humidity, motivating further investigation of whether there was still room for improvement by accounting for other covariates in the model.

### 3.4. Ozone Correction for LCS Calibration

**Figure 11:**
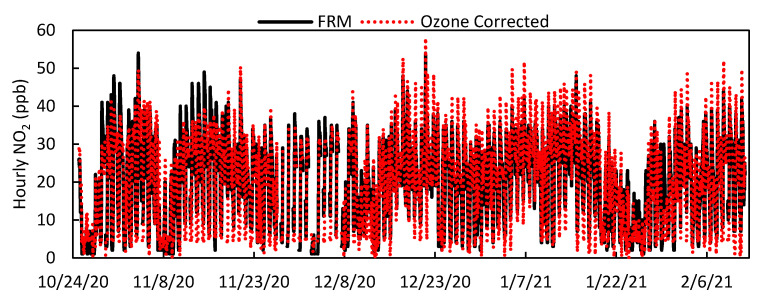
Time series of hourly West Phoenix FRM NO_2_ and LCS #2 Ozone corrected 30-day calibrated NO_2_ values for the entire deployment period.

**Figure 12:**
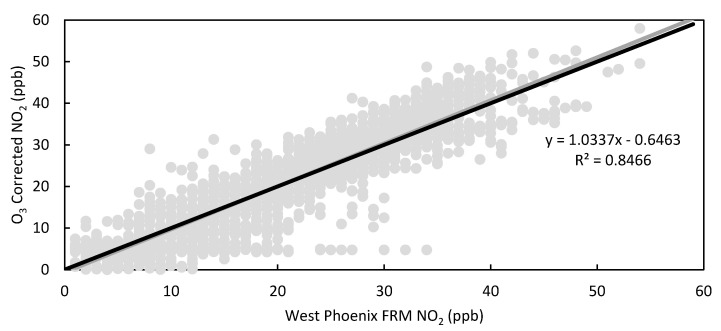
Scatter plot of hourly LCS #2 Ozone corrected 30-day calibration NO_2_ vs. West Phoenix FRM NO_2_ with trendline equation and R^2^, 1:1 line in black.

The Clarity calibration does not account for the effect of ozone on NO_2_ measurements because the LCS does not have a built-in O_3_ sensor even though the electrochemical sensor used to monitor for NO_2_ is known to also respond to O_3_ [26]. For this study, the LCSs were collocated at an air quality monitoring site that also had an O_3_ FEM instrument. In review of the Clarity calibrated data, an initial observation was that elevated ambient levels of ozone impacted LCS NO_2_ performance, motivating an ozone correction to data collected during high ambient ozone levels. This effect is illustrated in Figure 2. In addition to correcting for the influence of ozone, our supplemental calibration also was designed to eliminate negative values by establishing a baseline. Figure 11 and Figure 12 show the results of these corrections applied to the 30-day Clarity calibrated data on LCS #2. With this correction, the standard deviation between LCSs fell to 3.6 ppb, the RMSE decreased for all LCSs, and the R^2^ increased for all sensors as shown in Table 5. Compared to results of similar multivariate linear regression model studies [21], our data showed a higher average R^2^ (0.80) compared to (0.41), an average slope and intercept of (0.989, −0.3) compared to (0.664, 3.0), and a MAE of 4.6 ppb compared to 3.0 ppb.

**Figure 13:**
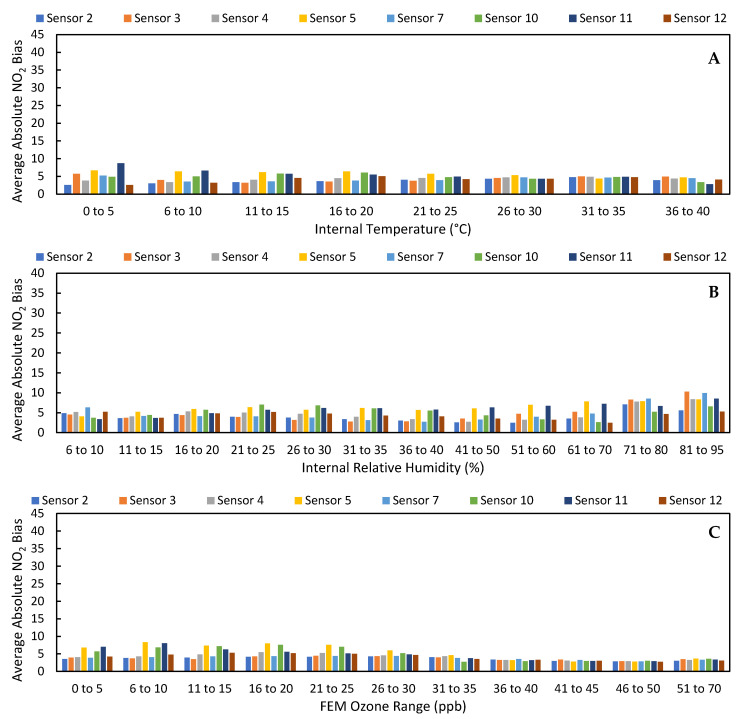
(**A**) The average absolute bias of the Ozone corrected 30-day LCS NO_2_ data binned by internal temperature values. (**B**) The average absolute bias of the raw LCS NO_2_ data binned by internal relative humidity values. (**C**) The average absolute bias of the raw LCS NO_2_ data binned by West Phoenix FEM O_3_ values.

**Figure 14:**
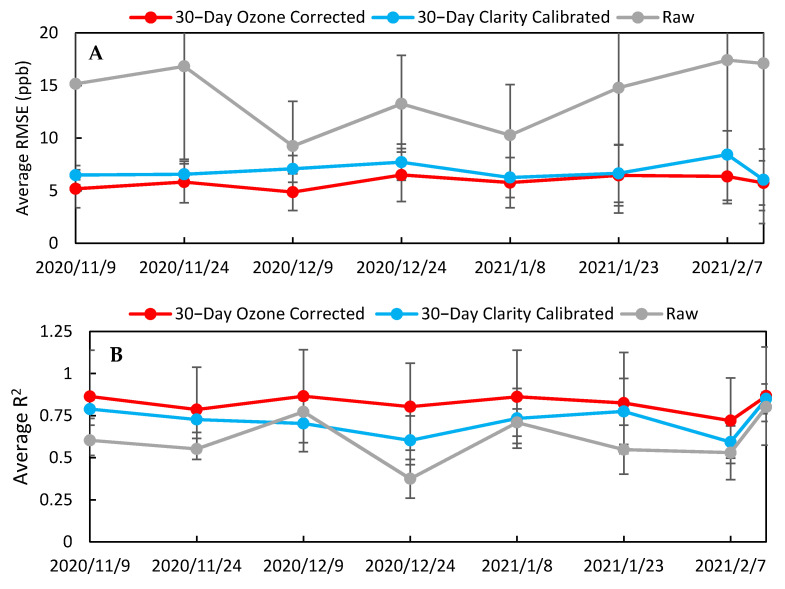
Average RMSE (**A**) and average R^2^ (**B**) between the 8 sensors evaluated biweekly for the whole deployment period for raw NO_2_, Clarity 30-day calibration, and Ozone corrected 30-day calibration.

In addition to improving sensor performance, this calibration also increased the LCS correlation to the FRM NO_2_ measurements (0.79 ≤ x ≤ 0.92) and optimized their response to environmental conditions compared to the 30-day Clarity calibration, as shown in Table S3. Furthermore, Figure 2 demonstrates that the impact of high ozone on the LCS NO_2_ measurements was lessened, as the LCS NO_2_/FRM NO_2_ values at ozone concentrations greater than 20 ppb were reduced. Figure 13 illustrates how the impact of environmental conditions on the sensor’s accuracy was reduced, as the absolute sensor biases decreased compared to Figure 5. Looking at the calibration’s performance over time in Figure 14, no temporal trends were observed; however, the ozone corrected data consistently had a RMSE that was lower and a R^2^ value that was higher than the Raw and Clarity calibrated data.

## 4. Conclusions

LCSs will continue to become more widespread as sensing technologies advance and the need for high-spatial-resolution air quality data grows. However, with this increased usage comes the necessity of ensuring the LCSs generate high data quality with an optimal approach for calibration and evaluation. This study showed that environmental factors such as relative humidity, temperature, and ozone can affect the raw measurements of these LCSs. However, by accounting for these variables in the LCS calibration, the accuracy and precision of the data can be improved. Additionally, this study demonstrated that increasing the calibration training data volume results in improved calibration performance. It is important to note that relatively steady environmental conditions throughout the deployment period benefited the performance of the LCSs; with rapidly changing environments such as extreme swings in temperature and relative humidity, one would anticipate more variable calibration performance and the need for more regular calibration adjustments. This study also showed the importance of developing a calibration model unique to each LCS, as not every LCS of a given brand and model responds to changing environmental conditions equally. This conclusion is based on the fact that two out of the eight tested sensors showed extraneous behavior demonstrating the importance of the scale of the study.

## Figures and Tables

**Table 1:** Results summary of NO_2_ LCSs tested by the South Coast Air Quality Management District [19].

Sensor Make (Model)	Field R^2^	Field MAE (ppb)
Aeroqual (AQY v0.5)	0.77	N/A
Aeroqual (AQY v1.0)	0.60–0.77	4.1–5.3
Airly	0.54–0.80	42.4–48.1
Air Quality Egg (Ver. 2)	0.0	N/A
APIS	0.30–0.44	6.1–9.4
AQMesh (V4.0)	0.0–0.46	N/A
AQMesh (V5.1)	0.49–0.54	7.6–8.4
CairPol (Cairsens NO_2_)	0.0–0.12	6.0–14.6
Igienair (Zaack AQI)	0.53–0.58	7.2–8.0
Kunak (Air A10)	0.24–0.32	6.6–7.4
Magnasci SRL (uRADMonitor INDUSTRIAL HW103)	0.00–0.05	11.6–24.8
Spec Sensors	0.0–0.16	N/A
Vaisala (AQT410 Ver. 1.11)	0.0	N/A
Vaisala (AQT410 Ver. 1.15)	0.43–0.61	13.0–16.3

**Table 2:** Data summary for the raw data from the 8 LCSs collocated at West Phoenix over the entire deployment period, calculated using West Phoenix FRM NO_2_ data.

Sensor	RMSE (ppb)	MAE (ppb)	R^2^	Slope [95% CI]
LCS #2	10.5	8.7	0.5974	1.13 [1.10–1.17]
LCS #3	9.9	8.1	0.5803	1.03 [0.99–1.06]
LCS #4	9.8	8.1	0.5951	1.05 [1.02–1.09]
LCS #5	32.7	26.6	0.3645	2.05 [1.95–2.15]
LCS #7	13.2	10.0	0.5223	1.17 [1.12–1.21]
LCS #10	6.7	5.4	0.6935	0.90 [0.87–0.92]
LCS #11	26.2	21.2	0.4039	1.79 [1.71–1.88]
LCS #12	8.0	6.7	0.6657	0.91 [0.88–0.93]

**Table 3:** Data summary for the Clarity 15-day calibration data from the 8 LCS collocated at West Phoenix over the entire deployment period, calculated using West Phoenix FRM NO_2_ data.

Sensor	RMSE (ppb)	MAE (ppb)	R^2^	Slope [95% CI]
LCS #2	7.0	5.4	0.7495	1.09 [1.06–1.11]
LCS #3	8.5	6.7	0.5810	0.85 [0.82–0.87]
LCS #4	8.5	6.6	0.6266	0.98 [0.95–1.01]
LCS #5	13.2	10.4	0.3485	0.76 [0.72–0.80]
LCS #7	8.0	6.2	0.6996	0.96 [0.94–0.99]
LCS #10	8.2	6.5	0.7426	1.07 [1.05–1.09]
LCS #11	13.2	10.6	0.3371	0.73 [0.69–0.77]
LCS #12	6.1	4.7	0.7953	1.08 [1.06–1.10]

**Table 4:** Data summary for the Clarity 30-day calibration data from the 8 LCSs collocated at West Phoenix over the entire deployment period, calculated using West Phoenix FRM NO_2_ data.

Sensor	RMSE (ppb)	MAE (ppb)	R^2^	Slope [95% CI]
LCS #2	5.3	4.0	0.7979	0.93 [0.91–0.95]
LCS #3	6.2	4.9	0.6893	0.75 [0.73–0.77]
LCS #4	6.2	4.8	0.7301	0.90 [0.88–0.92]
LCS #5	10.1	8.1	0.4300	0.69 [0.66–0.72]
LCS #7	6.6	5.0	0.6933	0.85 [0.83–0.87]
LCS #10	7.2	5.9	0.7637	0.98 [0.96–1.00]
LCS #11	9.2	7.7	0.4694	0.61 [0.59–0.64]
LCS #12	5.8	4.5	0.7956	0.97 [0.95–0.99]

**Table 5:** Data summary for the Ozone corrected 30-day calibration data from the 8 LCSs collocated at West Phoenix over the entire deployment period, calculated using West Phoenix FRM NO_2_ data.

Sensor	RMSE (ppb)	MAE (ppb)	R^2^	Slope [95% CI]
LCS #2	4.6	3.6	0.8466	1.04 [1.02–1.06]
LCS #3	5.0	3.8	0.8388	0.98 [0.97–1.00]
LCS #4	5.4	4.1	0.8337	1.07 [1.05–1.09]
LCS #5	7.9	6.1	0.6296	0.82 [0.80–0.84]
LCS #7	5.1	3.9	0.8200	0.96 [0.95–0.98]
LCS #10	6.8	5.3	0.8425	1.18 [1.16–1.20]
LCS #11	7.3	5.8	0.6966	0.76 [0.74–0.78]
LCS #12	5.4	4.2	0.8554	1.10 [1.08–1.12]

## Data Availability

Data supporting the reported results will be provided upon reader’s request.

## Supplementary Materials

The following are available online at https://www.mdpi.com/article/10.3390/toxics9110281/s1, Figure S1: MAE values for raw and Clarity 15-day calibrated LCS data, Figure S2: RMSE, MAE, R^2^, and slope comparisons for raw and Clarity 15-day calibrated data with training data excluded, Figure S3: Environmental factor comparison for the 15-day calibration period and the final 15 days of LCS deployment. Table S1: Slope and Pearson correlation coefficients of West Phoenix FRM NO_2_ and raw LCS data, Table S2: Slope and Pearson correlation coefficients of West Phoenix FRM NO_2_ and Clarity 30-day calibrated LCs data, Table S3: Slope and Pearson correlation coefficients of West Phoenix FRM NO_2_ and Ozone corrected 30-day calibrated LCs data.
