# Supplementary material for: Calibration of Low-Cost NO2 Sensors through Environmental Factor Correction
Source: Toxics. 2021 Oct 28;9(11):281. doi: 10.3390/toxics9110281 (PMC8624883; doi:10.3390/toxics9110281)
Supplement: Supplementary file 1 [file toxics-09-00281-s001.zip › toxics-1414773-supplementary.pdf]

toxics

MDPI

Supplementary Materials: Calibration of Low-Cost NO_2 Sensors through Environmental Factor Correction

Jason A. Miech, Levi Stanton, Meiling Gao, Paolo Micalizzi, Joshua Uebelherr, Pierre Herckes and Matthew P. Fraser

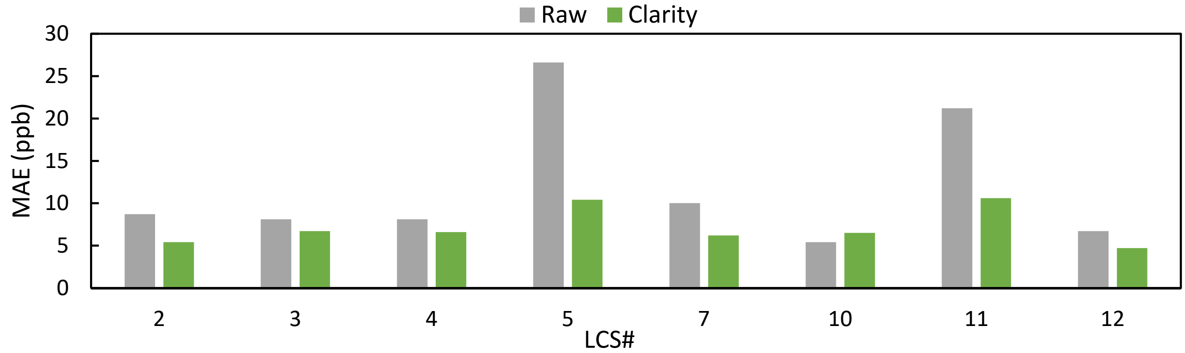

Figure S1. A comparison of the MAE (ppb) values between the raw and Clarity 15-day calibrated data.

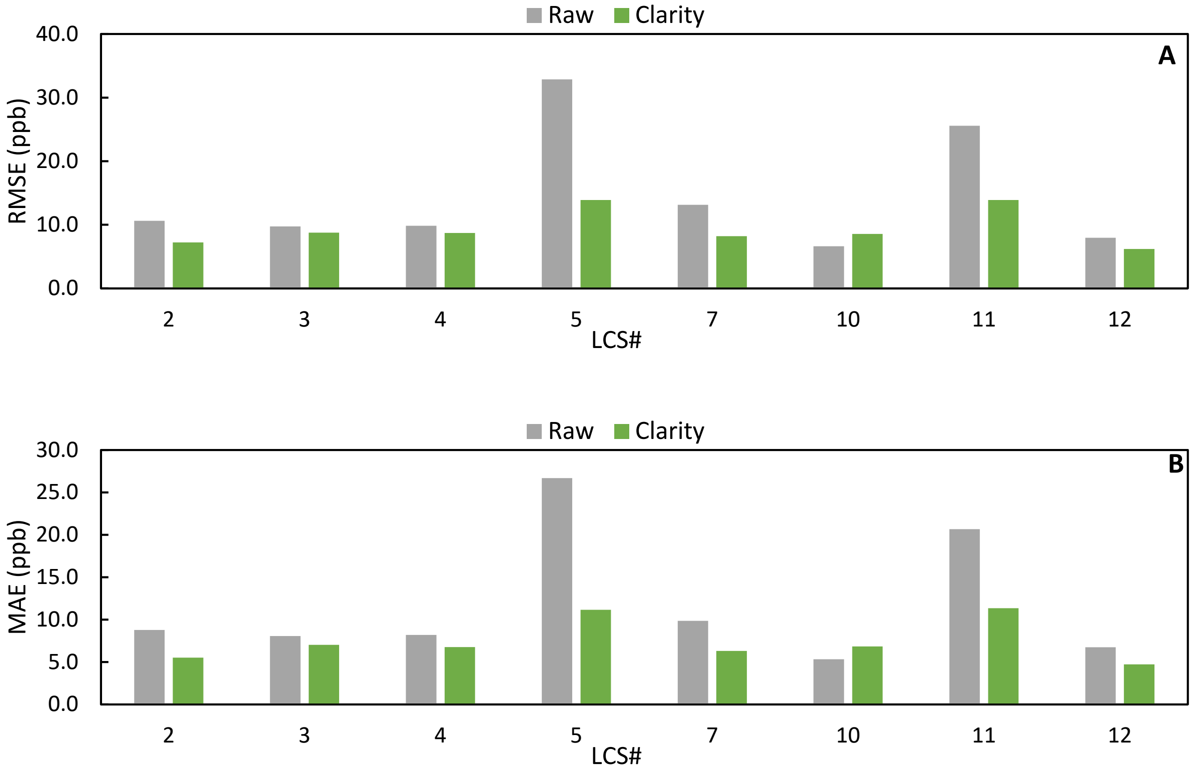

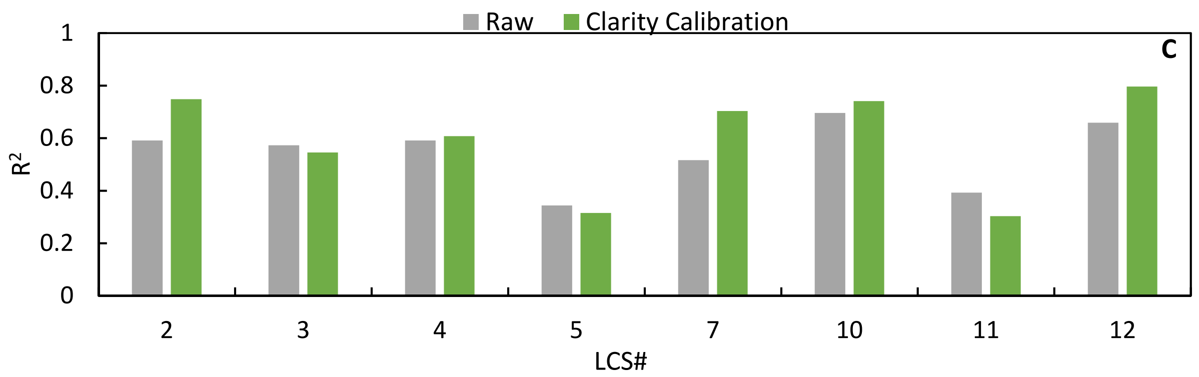

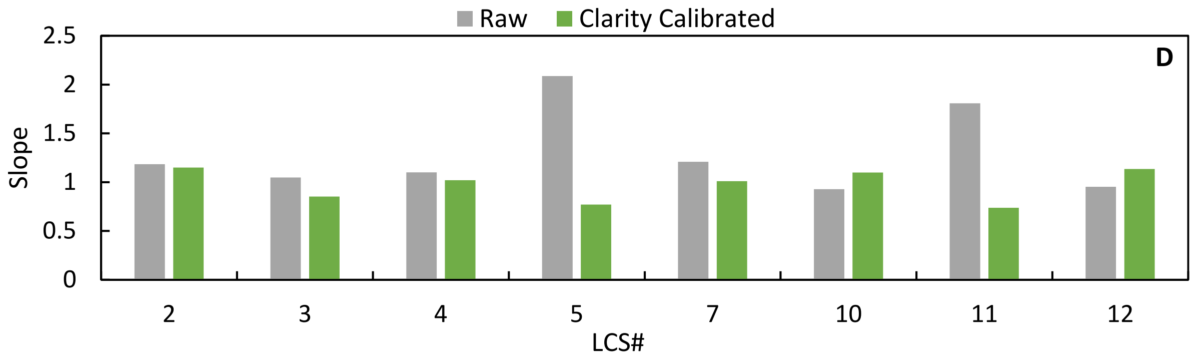

Figure S2. (**A**) A comparison of the RMSE (ppb) values between the raw and Clarity 15-day calibrated data with training data excluded. (**B**) A comparison of the MAE (ppb) values between the raw and Clarity 15-day calibrated data with training data excluded. (**C**) A comparison of the R^2 values between the raw and Clarity 15-day calibrated data with training data excluded. (**D)** A comparison of the slopes between the raw and Clarity 15-day calibrated data with training data excluded.

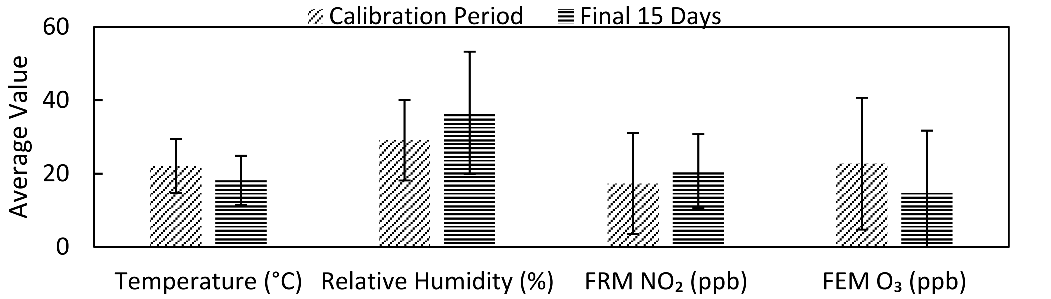

Figure S3. A comparison of the environmental factors experienced by LCS#2 during the 15-day calibration period and the final 15 days of deployment.

Table S1. Slopes and Pearson correlation coefficients of West Phoenix FRM NO_2 and raw data from the 8 LCSs for LCS internal temperature, LCS internal relative humidity, West Phoenix FRM NO_2, and West Phoenix FEM O_3. The West Phoenix FRM NO_2 slopes and correlation coefficients for temperature and relative humidity were calculated using the internal temperature and relative humidity from LCS #2.

	Temperature		Relative Humidity		FRM NO_2		FEM O_3	
	**Slope**	**Correlation Coeff.**	**Slope**	**Correlation Coeff.**	**Slope**	**Correlation Coeff.**	**Slope**	**Correlation Coeff.**
West Phoenix FRM NO_2	−0.48	−0.32	0.10	0.14	1.00	1.00	−0.56	−0.81
LCS #2	−1.43	−0.66	0.55	0.52	1.13	0.77	−0.89	−0.89
LCS#3	−1.24	−0.63	0.52	0.56	1.03	0.76	−0.81	−0.88
LCS#4	−1.23	−0.62	0.51	0.54	1.05	0.77	−0.82	−0.88

LCS#5	−3.03	−0.62	1.26	0.59	2.05	0.60	−1.82	−0.78
LCS#7	−1.7	−0.72	0.69	0.61	1.17	0.72	−0.99	−0.89
LCS#10	−0.95	−0.61	0.36	0.48	0.90	0.83	−0.66	−0.90
LCS#11	−2.64	−0.63	1.09	0.59	1.80	0.64	−1.56	−0.81
LCS #12	−0.96	−0.59	0.38	0.48	0.91	0.82	−0.69	−0.89

Table S2. Slopes and Pearson correlation coefficients of West Phoenix FRM NO_2 and Clarity 30-day calibrated data from the 8 LCSs for LCS internal temperature, LCS internal relative humidity, West Phoenix FRM NO_2, and West Phoenix FEM O_3. The West Phoenix FRM NO_2 slopes and correlation coefficients for temperature and relative humidity were calculated using the internal temperature and relative humidity from LCS #2.

	**Temperature**		**Relative Humidity**		**FRM NO_2**		**FEM O_3**	
	**Slope**	**Correlation Co-eff.**	**Slope**	**Correlation Co-eff.**	**Slope**	**Correlation Co-eff.**	**Slope**	**Correlation Co-eff.**
West Phoenix FRM	−0.48	−0.32	0.10	0.14	1.00	1.00	−0.56	−0.81
LCS #2	−0.46	−0.30	0.02	0.03	0.93	0.89	−0.53	−0.73
LCS#3	−0.17	−0.13	−0.09	−0.15	0.75	0.83	−0.36	−0.59
LCS#4	−0.36	−0.23	−0.03	−0.04	0.90	0.85	−0.48	−0.67
LCS#5	−0.36	−0.23	0.07	0.10	0.69	0.66	−0.44	−0.60
LCS#7	−0.35	−0.24	−0.003	−0.01	0.85	0.83	−0.46	−0.66
LCS#10	−0.43	−0.26	−0.05	−0.07	0.98	0.87	−0.52	−0.67
LCS#11	−0.14	−0.11	−0.05	−0.08	0.61	0.69	−0.34	−0.55
LCS #12	−0.47	−0.30	0.05	−0.06	0.97	0.89	−0.55	−0.73

Table S3. Slopes and correlation coefficients of West Phoenix FRM NO_2 and Ozone corrected 30-day calibrated data from the 8 LCSs for LCS internal temperature, LCS internal relative humidity, West Phoenix FRM NO_2, and West Phoenix FEM O_3. The West Phoenix FRM NO_2 slopes and correlation coefficients for temperature and relative humidity were calculated using the internal temperature and relative humidity from LCS #2.

	**Temperature**		**Relative Humidity**		**FRM NO_2**		**FEM O_3**	
	**Slope**	**Correlation Coeff.**	**Slope**	**Correlation Coeff.**	**Slope**	**Correlation Coeff.**	**Slope**	**Correlation Coeff.**
West Phoenix FRM	−0.48	−0.32	0.10	0.14	1.00	1.00	−0.56	−0.81
LCS #2	−0.59	−0.36	0.09	0.11	1.03	0.92	−0.61	−0.80
LCS#3	−0.44	−0.28	0.04	0.06	0.98	0.92	−0.55	−0.75
LCS#4	−0.54	−0.32	0.07	0.08	1.07	0.91	−0.62	−0.77
LCS#5	−0.48	−0.32	0.14	0.21	0.82	0.79	−0.52	−0.74
LCS#7	−0.45	−0.29	0.05	0.06	0.96	0.91	−0.55	−0.75
LCS#10	−0.67	−0.35	0.07	0.08	1.18	0.92	−0.69	−0.78
LCS#11	−0.33	−0.24	0.06	0.10	0.76	0.83	−0.45	−0.72
LCS #12	−0.60	−0.35	0.11	0.13	1.10	0.92	−0.66	−0.80
